# Long-term effects of simulated microgravity in the central nervous system of rhesus monkeys: a voxel-wise multimodal MRI study

**DOI:** 10.3389/fphys.2025.1634366

**Published:** 2025-10-24

**Authors:** Jiarui Wang, Dong Zheng, Chenhao Zhao, Xuan Xuan, Xiaoyun Zhang, Ruyue Li, Kai Xu, Yawei Zeng, Youping Tao, Dongxu Lu, Li Liu, Suping Zhao, Yanli Liu, Jigong Wu, Juan Du

**Affiliations:** ^1^ Department of Neurology, The Ninth Medical Center, Chinese PLA General Hospital, Beijing, China; ^2^ PLA 306 Clinical College, Anhui Medical University, Beijing, China; ^3^ The Fifth Medical college, Anhui Medical University, Beijing, China; ^4^ Department of Imaging, The Ninth Medical Center, Chinese PLA General Hospital, Beijing, China; ^5^ The Second Affiliated Hospital of Anhui Medical University, Hefei, Anhui, China; ^6^ Department of Spine Surgery, The Ninth Medical Center, Chinese PLA General Hospital, Beijing, China

**Keywords:** simulated microgravity, structural MRI, diffusion tensor imaging, functional MRI, longitudinal study

## Abstract

**Background:**

With the development of spaceflight, scientists have gradually realized that long-term microgravity can alter the brain’s structure, which may affect the stability of brain function and, in turn, cognition and many other behaviors.

**Objective:**

By quantitatively analyzing the effects of microgravity on brain gray matter volume, fiber tracts, and resting-state neural functional activity, this study preliminarily explores the dynamic changes in brain tissue structure and their relationships during simulated microgravity.

**Methods:**

Six male rhesus macaques were included in the study and underwent −10° head-down bed rest (HDBR) for 42 days as a terrestrial analog of the microgravity environment. Multimodal magnetic resonance imaging (MRI) was performed 3 days before HDBR, 21 days after HDBR, and 42 days after HDBR. Voxel-based morphometry (VBM) analysis was used to compare differences in brain gray matter volume. Differences in the fractional anisotropy (FA), mean diffusivity (MD), axial diffusivity (AD), and radial diffusivity (RD) were investigated using tract-based spatial statistic (TBSS) analysis. Resting-state functional MRI was used to compare differences in local neural activity.

**Results:**

During simulated microgravity, significant changes in gray matter volume were found in the right substantia innominate of the basal forebrain, right insula, left putamen, and left occipital gyrus. A significant decrease in FA and AD was found during simulated microgravity, specifically in the left inferior longitudinal fasciculus, left fornix, left corticospinal tract, left inferior longitudinal fasciculus, left superior longitudinal fasciculus, left frontal aslant tract, right uncinate fasciculus, and bilateral inferior fronto-occipital fasciculus regions. A significant decrease in MD and RD was widely observed in the left inferior longitudinal fasciculus, middle cerebellar peduncle, bilateral frontal aslant tract regions, bilateral anterior thalamic radiation, and bilateral uncinate fasciculus. Regional homogeneity (ReHo) in the left thalamic reticular nucleus continuously increased during simulated microgravity conditions.

**Conclusion:**

Using multimodality MRI, this study indicated that simulated microgravity might cause widespread abnormalities through neuroplasticity, especially in brain regions in charge of visuospatial awareness and voluntary motion. There may exist a complex functional compensation between the reconstruction of gray and white matter and the rearrangement of neural connections.

## 1 Introduction

In recent years, scientists have focused on central nervous system changes induced by microgravity during human spaceflight. Astronauts are confronted to multiple spaceflight-associated stressors, especially a dramatic decrease in gravity force. However, in the real world, it is very hard to selectively discriminate the strict role of microgravity on physiological, and particularly cerebral, dysfunction (Moore et al., 2019; Porte and Morel, 2012). Thus, ground-based models are a suitable alternative to space microgravity and could induce similar modifications in the central nervous system. The head-down tilt bed rest (HDBR) model, as a golden standard analog for microgravity, effectively simulates fluid redistribution and mechanical loading to the hindlimbs, which provides a similar condition as microgravity (Moore et al., 2010). This approach replicates key physiological conditions observed in spaceflight, including cephalad fluid shifts, reduced weight-bearing on hindlimbs, and musculoskeletal degeneration. Its validity is further evidenced by hemodynamics (Roberts et al., 2021), neurocognitive network (Hargens and Vico, 2016), and molecular pathways (Zhang et al., 2019; Lin et al., 2020). The HDBR model in rhesus monkeys was selected because the central nervous system of primates is most similar to that of humans compared with other species (Convertino et al., 1998).

Notably, one of the commonly reported neurological effects of microgravity is that the brain largely “floats upward,” causing a salient redistribution of cerebrospinal fluid (CSF) (Roberts et al., 2017), followed by remodeling of gray and white matter (Seidler et al., 2024). In recent years, with the continuous development and innovation of multimodal magnetic resonance imaging (MRI) technology in the medical field, significant breakthroughs have been achieved in the study of microgravity-related changes in the central nervous system. Through this technology, we have gradually realized that the structural changes in the brain inevitably contribute to the stability of brain function and affects cognition and many other behaviors. However, to the best of our knowledge, there is limited research regarding the joint analysis and process of the longitudinal effects of microgravity on the brain structure.

To determine changes in brain structural plasticity under simulated microgravity, the current study aimed to investigate the MRI-based characteristics of rhesus monkeys before, during, and after prolonged exposure to HDBR.

## 2 Materials and methods

### 2.1 Animals

This study uses a within-subject control design to minimize individual differences and reduce sample size requirements. A total of six male rhesus macaques (*Macaca mulatta*) of Chinese origin, from the China Astronaut Research and Training Center, were utilized in our longitudinal study. All animals were confirmed to be healthy, and control of viruses, bacteria, parasites, and other pathogens complied with the national standards for ordinary-grade nonhuman primates. Before being assigned to the project, all animals were adapted for 38 days in the barrier facility of the animal laboratory. All animals were 4–8 years old, 85–95 cm in length, and weighed 6–10 kg. The serial numbers of the animals were 195001, 165007, 185001, 180605, 175005, and 185009.

The HDBR model was used as a microgravity analog in terrestrial rhesus monkeys. The head-down animals were placed on beds, which were tilted 10° backward from the horizontal. The rhesus monkeys in the head-down position were secured to the bed using a specialized cloth, which was humanely attached to the bed frame of the HDBR platform. The specialized cloth covered the elbows and knees to prevent skin abrasions from friction with the device, while two forearms and lower legs were not fixed to the bed. This setup limited major body movement and maintained the −10° head-down tilt position, while still allowing forelimb and hindlimb horizontal motion to free access to food, water, and fruits. Animals received a standardized diet and were individually housed in temperature-controlled rooms (23 °C ± 2 °C) under a 12:12-h light–dark cycle (lights on: 08:00; lights off: 20:00). The entire process was continuously supervised and monitored 24 h a day. Environmental enrichment included provision of toys, which were available all the time, except during MRI scanning procedures. Animal caretakers provided daytime companionship to reduce anxiety and tracked body weight every week to monitor animal health. More details were provided in our previous work (Sun et al., 2017). All animal experimental protocols involved in this study were approved by the Experimental Animal Management and Use Committee of the China Astronaut Research and Training Center (Approval No. ACC-IACUC-2024-024), which is in accordance with the principles of the Association for Assessment and Accreditation of Laboratory Animal Care International (AAALAC), approved by the Institutional Animal Care and Use Committee (IACUC). All animal housing occurred in the Animal Facility at China Astronaut Research and Training Center.

### 2.2 MRI scanning

MRI scans were acquired prior to HDBR as the baseline (Time 1, T1), and at the third (Time 2, T2) and sixth weeks (Time 3, T3) post-HDBR. All scans were performed using a Siemens Verio 3.0T magnetic resonance scanner (Siemens, Germany) with a 32-channel head coil at the Ninth Medical Center of PLA General Hospital. Structural MRI, diffusion-weighted MRI, and resting-state functional MRI were performed under anesthesia induced by intramuscular injection of ketamine hydrochloride (7.5 mg/kg, Jiangsu ZhongMu BeiKang Pharmaceutical CO., LTD) and xylazine (5 mg/kg, Changsha Best Biological Technology Institute CO., LTD). Anesthesia remained stable for at least 1 h, and the scan duration was less than 1 h. Fingertip blood oxygen and heart rate detectors were used to monitor basic vital signs. Veterinarians and investigators observed the animal conditions through the glass of the scanning room. MRI scans were performed 3 days prior to HDBR (T1) and in 21 days (T2) and 42 days (T3) post-HDBR. T1 weighted images were acquired with a three-dimensional (3D) T1 magnetization-prepared rapid gradient-echo (MPRAGE) sequence. The parameters were as follows: repetition time/echo time (TR/TE) = 2,300/3.49 ms, FOV = 140 mm × 140 mm, data matrix = 256 × 256, flip angle = 10°, and slice thickness = 1 mm (voxel size = 0.5 × 0.5 × 1.0 mm^3^). The scan duration was 6 min 56 s. A ep2d-diff-mddw sequence in the axial orientation was used in the DTI scans. Diffusion-weighted images (DWIs) were acquired along 30 noncollinear and noncoplanar directions, with b = 1,000 s/mm^2^ and 1 b = 0 s/mm^2^ image. The parameters were as follows: TR/TE = 10,000/98 m, FOV = 191 mm × 191 mm, data matrix = 130 × 130, flip angle = 90°, and 19 slices with 3 mm slice thickness (voxel size = 1.5 × 1.5 × 3.0 mm^3^). The scan duration was 10 min 52 s. Resting-state functional MRI images were acquired with the ep2d-pace-dynat sequence with the following parameters: TR/TE = 2,500/29 m, FOV = 140 mm × 140 mm, data matrix = 64 × 640, flip angle = 90°, and slice thickness = 3 mm (voxel size = 2.2 × 2.2 × 3.0 mm^3^). The scan duration was 15 min 7 s.

### 2.3 VBM

Preprocessing for VBM was carried out in SPM12 and CAT12 running on MATLAB (Mathworks Inv., Sherborn, MA, United States). Since the tissue probability maps (TPMs) implemented in SPM12 were for humans, we used the macaque TPMs provided by Rohlfing et al. (2012) and segmented the images into grey matter (GM), white matter (WM), and cerebrospinal fluid (CSF). Each segmented image was used to create the investigated monkey GM, WM, and CSF templates based on the diffeomorphic anatomical registration through exponentiated lie algebra (DARTEL) algorithm. Subsequently, the individual GM images were spatially normalized to the investigated monkey templates. The normalized images were then registered to the INIA19 macaque brain template using affine parameter transformations and resliced to a voxel size of 1.5 × 1.5 × 1.5 mm. The voxel values were modulated by multiplying the GM images by Jacobian determinants of the deformation field to allow for comparison of the absolute amount of the tissue corrected for individual GM volumes. The resulting images were then smoothed with an isotropic Gaussian kernel of 6 mm full width at half maximum.

### 2.4 DTI data analysis

MRtrix3.0.4 (https://www.mrtrix.org/) software was used for data preprocessing. First, all raw DWI volumes were aligned to the b0 image, and the background noise and ring artifacts were removed using MRtrix tools. Then, head motion and eddy current distortions were corrected using the dwifslpreproc command, which integrates functions from the FMRIB Software Library (FSL, https://fsl.fmrib.ox.ac.uk/fsl/fslwiki/) (Smith et al., 2004). B-vectors were adjusted accordingly. To correct for intensity inhomogeneities from the MRI scanner, N4 bias field correction was applied using the ANTs toolkit. Skull stripping and removal of non-brain tissues were performed manually. Finally, voxel-wise diffusion tensor modeling was performed using the FSL dtifit tool (Basser et al., 1994), yielding maps of fractional anisotropy (FA), mean diffusivity (MD), axial diffusivity (AD), and radial diffusivity (RD) derived from the eigenvalues of the diffusion tensor.

Subsequent analysis used tract-based spatial statistic (TBSS) to identify major WM tracts. Voxel-wise analysis on FA images was conducted using the TBSS toolbox of FSL (Smith et al., 2004), modified according to established protocols for nonhuman primate (NHP) neuroimaging (Tang et al., 2016). Each FA image was nonlinearly registered to an individual’s T1 template, followed by transformation into the D99 Macaque Stereotaxic Atlases (Saleem et al., 2021) with 1 × 1 × 1 mm^3^ resolution. Individual FAs were converted to the standard space, and a mean FA image was generated across all subjects. Then, the mean FA image was skeletonized, and a mean FA skeleton was derived using a threshold of 0.2 to exclude small peripheral tracts and retain the major WM tracts common to all the subjects. Each subject’s FA maps were projected onto this skeleton by filling the voxel values from the nearest tract center. The skeletonized FA images were included in group-level statistical analyses. The tbss_nonFA commands were applied to project MD, AD, and RD onto the same FA skeleton.

### 2.5 fMRI analysis

The preprocessing was implemented using the DPABI toolbox for the monkey module (Yan and Zang, 2010). Preprocessing steps included the removal of the first 10 time-point volumes, field map correction, slice timing and motion correction, normalization of standard space of the monkey D99 atlas (Saleem et al., 2021), 2-mm cubic voxel resampling, linear detrending, nuisance covariate regression, and band-pass filtering.

Kendall’s coefficient of concordance (KCC) was used to measure regional homogeneity or similarity of the ranked time series of a given voxel with its nearest 26 neighbor voxels in a voxel-wise way. Then, Dpabi was used to calculate the KCC value of the time series of a given voxel with those of its nearest neighbors (26 voxels) in a voxel-wise analysis. To normalized regional homogeneity (ReHo) maps, we divided each voxel's KCC value by the global mean KCC. Then, the ReHo maps were spatially smoothed with 3-mm FWHM Gaussian kernel.

### 2.6 Statistical analysis

Statistical analysis and visualization were performed using MATLAB 2022b, FSL, and GraphPad Prism 10. No adverse events were observed during the experiments. So, each analysis has the same sample size (n = 6).

For VBM, we performed voxel-by-voxel repeated-measure ANOVA to make group-level statistical comparison of GM changes across different time points. Total intracranial volume (TIV) was set as a covariate in all analyzed studies. We selected a threshold of P < 0.01 (uncorrected for multiple comparisons) at the voxel level across the whole brain, with a cluster size of 10 voxels. No statistically significant results were found after FWE correction (P_FWE_ < 0.05). The *post hoc* analysis used paired t-test to investigate the time course of the GM changes in the regions.

To reveal the WM impairments due to microgravity, the three group comparisons were conducted with pairwise t-test on the skeletonized FA images from the DTI scans. A nonparametric general linear model using a randomized tool in FSL (Winkler et al., 2014) was applied to perform paired group comparisons with 5,000 permutations and threshold-free cluster-enhancement (TFCE) (Smith and Nichols, 2009) to correct for multiple comparisons. Statistically significant outcomes were considered at FWE-corrected P_FWE_ < 0.05. The differences in FA, AD, RD, and MD values were calculated separately. The differential brain regions were reported based on XTRACT Macaque Probabilistic Tract Atlases (Warr et al., 2020) and displayed on the D99 Macaque Atlases (Saleem et al., 2021).

To explore the ReHo differences during simulated microgravity conditions, repeated-measure ANOVA was performed on the normalized ReHo maps. The resultant statistical map was set at a combined threshold of P < 0.001 and a minimum cluster size of 10 voxels, corresponding to a corrected threshold of P < 0.05. Statistically significant outcomes were considered at FWE-corrected P_FWE_ < 0.05. The *post hoc* analysis used paired t-test to investigate the time course of the GM changes in the regions. The differential brain regions were displayed based on D99 Macaque Atlases (Saleem et al., 2021).

## 3 Results

### 3.1 General description

**FIGURE 1:**
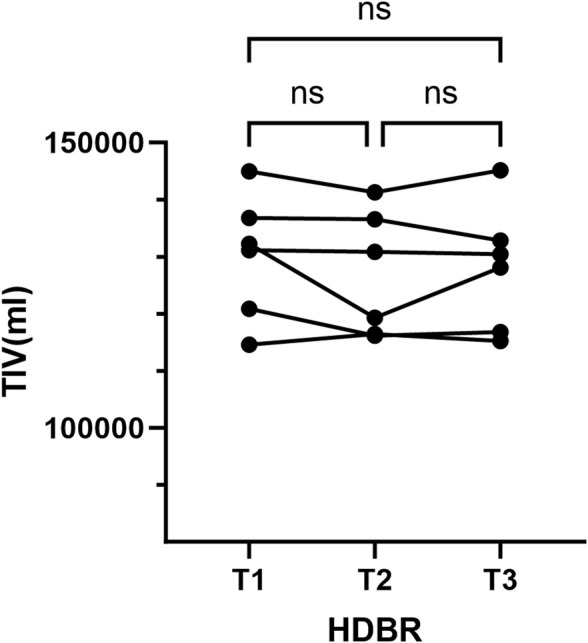
Total brain volume measurements. ns, no significance; T1, pre-HDBR; T2, 21 days of HDBR; T3, 42 days of HDBR.

No lesions were found in any of the scans. We calculated the TIV based on individual de-skulled structural images. The repeated-measure ANOVA showed that there were no significant changes in TIV during HDBR (Figure 1).

### 3.2 Changes in gray matter volume after HDBR

**FIGURE 2:**
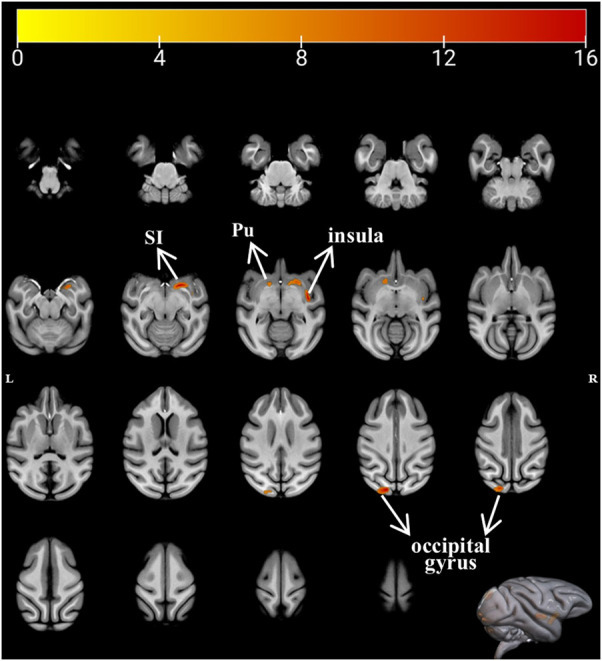
Axial brain slices showing difference in GM volume after HDBR. R, right; L, left; Pu, putamen; SI, substantia innominate.

**FIGURE 3:**
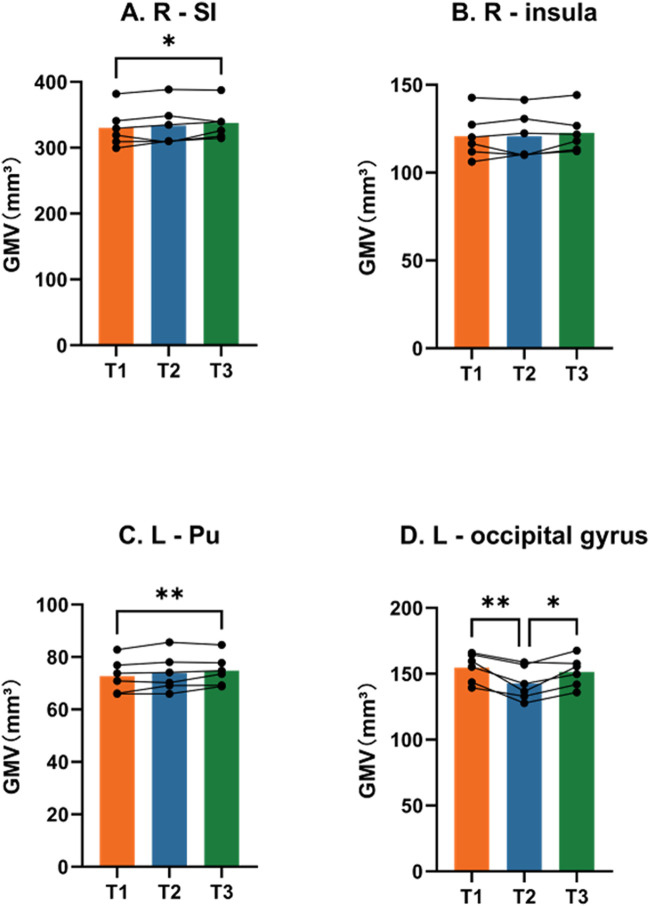
*Post hoc* pairwise comparisons. Longitudinal changes in the mean GMV in clusters. *, P < 0.05; **, P < 0.01; R, right; L, left; Pu, putamen; SI, substantia innominate.

We performed VBM analysis to identify the structural changes that occurred during simulated microgravity conditions. Results were displayed in the T1 template from the INIA19 rhesus atlas (Rohlfing et al., 2012). Figure 2 and Table 1 show the regions with significant alterations in GM volume using repeated-measure ANOVA (P < 0.01, uncorrected, cluster size >10). Significant voxels were observed in the right substantia innominate (r-SI) of the basal forebrain (BF) area, right insula, left putamen (l-Pu), and left occipital gyrus. In our samples, the multiple comparison correction failed to detect any significant voxels. *Post hoc* pairwise comparisons demonstrated a statistically significant increase in r-SI (Figure 3A, P = 0.0470) and l-Pu (Figure 3C, P = 0.0063) from 6 weeks after HDBR compared to that in pre-HDBR. The GM in the left occipital gyrus significantly decreased (Figure 3D, T1 vs. T2, P = 0.0132) and then dramatically increased to the normal level (Figure 3D, T2 vs. T3, P = 0.0448; T1 vs. T3, P = 0.3185).

**TABLE 1:** Significant clusters displaying an alternation in GM volume after HDBR.

Estimated area	Peak			F value	Cluster size (voxel)	GMV (mm^3^)		
	X	Y	Z			T1	T2	T3
R-SI	8.5	2	−4.5	21.8478	136	330.01 ± 26.68	333.34 ± 28.87	337.42 ± 24.34
R-insula	17.5	−6	−1.5	20.2225	49	120.81 ± 11.77	120.81 ± 12.03	122.69 ± 10.82
L-Pu	−7.5	4	−0.50	10.7053	29	72.71 ± 5.95	73.84 ± 6.50	74.80 ± 5.40
L-occipital gyrus	−6.5	−38	12.5	19.1282	109	154.67 ± 10.05	142.56 ± 11.68	151.40 ± 10.43

R, right; L, left; Pu, putamen; T1, pre-HDBR; T2, 21 days of HDBR; T3, 42 days of HDBR; SI, substantia innominate.

### 3.3 Changes in the white matter fiber after HDBR

Comparing to baseline, monkeys after 21 days (Table 2) and 42 days of HDBR (Table 3) presented with significantly decreased FA, MD, AD, and RD. Figures illustrate the results in the T1 template from the D99 rhesus atlas (Goldman-Rakic and Rakic, 1991).

**TABLE 2:** White matter with decreased FA, MD, AD, or RD after 21 days of HDBR (T1 > T2, P_FWE_ < 0.05).

Cluster number	Cluster size (voxels)	Brain region	Peak of mass			TBSS metrics	Function
			X	Y	Z		
1	332	L-ILF	−11.8	−5.83	−9.17	FA	Memory and emotion
		L-FX					Memory
2	5,042	L-SLF	−13.8	7.17	−2.17	MD	Visuospatial awareness
		MCP					Motor coordination
		BIL-FAT					Execution and language
3	1,393	L-CST	−17.8	13.2	3.83	AD	Voluntary motion
		L-IFO					Visuospatial awareness
		L-ILF					Memory and emotion
4	126	R-IFO	16.2	8.17	3.83	AD	Visuospatial awareness
		R-UF					Emotion
5	363	MCP	−6.78	−37.8	−10.2	RD	Motor coordination
6	260	BIL-ATR	−2.78	−10.8	−2.17	RD	Emotion
7	253	L-UF	−14.8	8.17	−2.17	RD	Emotion and language

L, left; R, right; BIL, bilateral; ILF, inferior longitudinal fasciculus; FX, fornix; SLF, superior longitudinal fasciculus; MCP, middle cerebellar peduncle; FAT, frontal aslant tract; CST, cortico-spinal tract; IFO, inferior fronto-occipital fasciculus; UF, uncinate fasciculus; ATR, anterior thalamic radiation; FA, fractional anisotropy; MD, mean diffusivity; AD, axial diffusivity; RD, radial diffusivity; T1, pre-HDBR; T2, 21 days of HDBR.

**TABLE 3:** White matter with decreased FA, MD, AD, or RD after 42 days of HDBR (T1 > T3, P_FWE_ < 0.05).

Cluster number	Cluster size (voxel)	Brain region	Peak of mass			TBSS metrics	Function
			X	Y	Z		
1	425	L- ILF	−18.8	−4.83	−9.17	FA	Memory and emotion
2	2,508	BIL-FAT	−7.78	4.14	3.83	MD	Execution and emotion
		BIL-ATR					Emotion
		BIL-UF					Emotion and language
3	629	MCP	−5.78	−36.8	−9.17	MD	Motor coordination
4	376	L-SLF	−14.8	8.17	6.83	AD	Visuospatial awareness
		L-FAT					Language
5	221	R-FAT	8.22	14.2	16.8	RD	Execution
6	216	L-UF	−15.8	2.17	−10.2	RD	Emotion and language

L, left; R, right; BIL, bilateral; ILF, inferior longitudinal fasciculus; FAT, frontal aslant tract; ATR, anterior thalamic radiation; UF, uncinate fasciculus; MCP, middle cerebellar peduncle; SLF, superior longitudinal fasciculus; FA, fractional anisotropy; MD, mean diffusivity; AD, axial diffusivity; RD, radial diffusivity; T1, pre-HDBR; T3, 42 days of HDBR.

**FIGURE 4:**
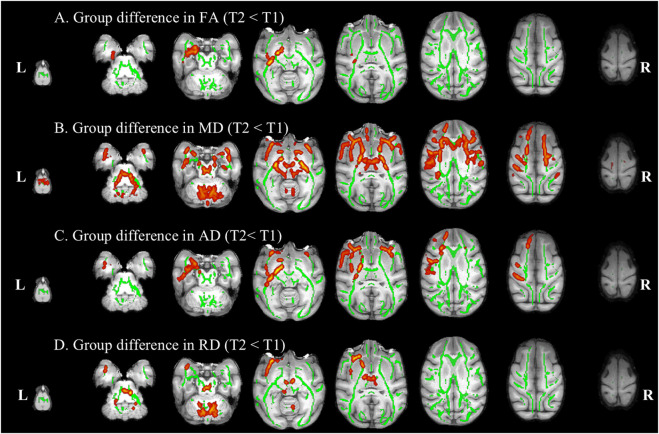
Compared with baseline, white matter difference after 21 days of HDBR. White matter regions (warm color: yellow–red) showed decreased FA **(A)**, MD **(B)**, AD **(C)**, and RD **(D)** values in monkeys after 21 days of HDBR, compared to pre-HDBR (PFWE <0.05). The white matter regions with green color represent the FA skeleton. R, right; L, left; T1, pre-HDBR; T2, 21 days of HDBR.

**FIGURE 5:**
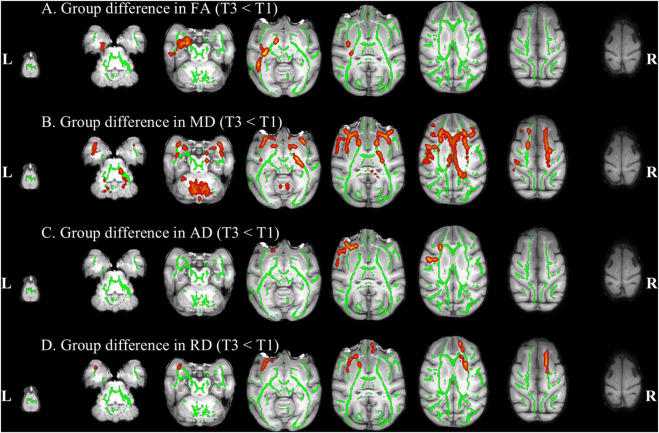
Compared with baseline, white matter difference after 42 days of HDBR. White matter regions (warm color: yellow–red) showed decreased FA **(A)**, MD **(B)**, AD **(C)**, and RD **(D)** values in monkeys after 42 days of HDBR (down), compared to pre-HDBR (PFWE <0.05). The white matter regions with green color represent the FA skeleton. R, right; L, left; T1, pre-HDBR; T3, 42 days of HDBR.

Compared to pre-HDBR, the decrease in FA was detected in the left inferior longitudinal fasciculus and left fornix region, and a decrease in AD was observed in the left corticospinal tract, left inferior longitudinal fasciculus, right uncinate fasciculus, and bilateral inferior fronto-occipital fasciculus regions after 21 days of simulated microgravity conditions (Figures 4A,C). After 42 days of simulated microgravity, the regions showing decreased FA had expanded and remained in the left inferior longitudinal fasciculus regions. Meanwhile, a decrease in AD was observed in the left superior longitudinal fasciculus and frontal aslant tract regions (Figures 5A,C). These changes suggest destruction in white matter integrity in regions associated with memory, emotion, visuospatial awareness, and motion.

Compared to pre-HDBR, a widespread decrease in MD was observed in the left inferior longitudinal fasciculus, middle cerebellar peduncle, and bilateral frontal aslant tract regions; in addition, a decrease in RD was detected in the middle cerebellar peduncle, right anterior thalamic radiation, and left uncinate fasciculus regions after 21 days of simulated microgravity (Figures 4B,D). After 42 days of simulated microgravity, the regions with decreased MD and RD were reduced. A decrease in MD was observed in the middle cerebellar peduncle, bilateral frontal aslant tract, bilateral anterior thalamic radiation, and bilateral uncinate fasciculus regions. A decrease in RD was detected in the right frontal aslant tract and left uncinate fasciculus regions (Figures 5B,D). These changes indicate that potential optimization of white matter microstructure, such as increased fiber density and myelination, may partially compensate for the impairment in white matter integrity. Moreover, the compensatory fibers may reduce with the extension of simulated microgravity.

**FIGURE 6:**
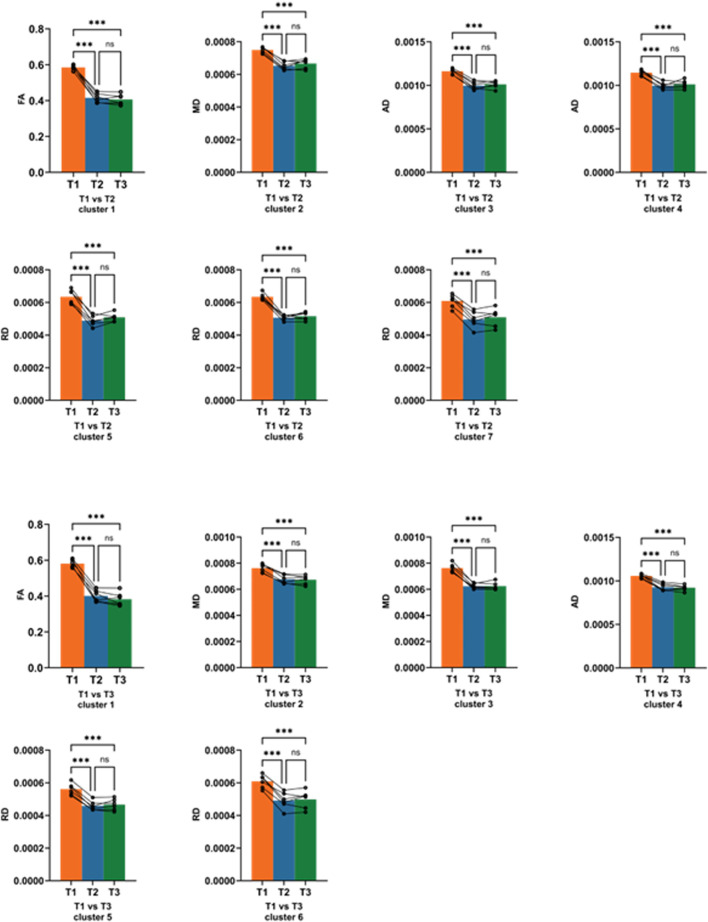
*Post hoc* analysis of white matter difference among pre-HDBR and 21 and 42 days post-HDBR. ns, no significance; ***, P < 0.001; T1, pre-HDBR; T2, 21 days of HDBR; T3, 42 days of HDBR. **(A)** Group difference in FA (T3 < T1). **(B)** Group difference in MD (T3 < T1). **(C)** Group difference in AD (T3 < T1). **(D)** Group difference in RD (T3 < T1).

However, when comparing HDBR at 21 and 42 days of simulated microgravity, no significant changes were observed in any tensor metrics, indicating that alterations in white matter fiber tracts may mainly occur at an early stage of simulated microgravity conditions. Values of every different brain region at three time points were subjected to *post hoc* analysis. Paired t-tests revealed that the decreases in values were already significantly observed at 21 days of HDBR (Figure 6). The results of the *post hoc* analysis suggest that both 21 and 42 days of simulated microgravity conditions caused alterations in all the mentioned brain regions in rhesus monkeys, and the significant changes are likely to occur at an earlier stage of simulated microgravity.

### 3.4 Changes in the functional activity after HDBR

**FIGURE 7:**
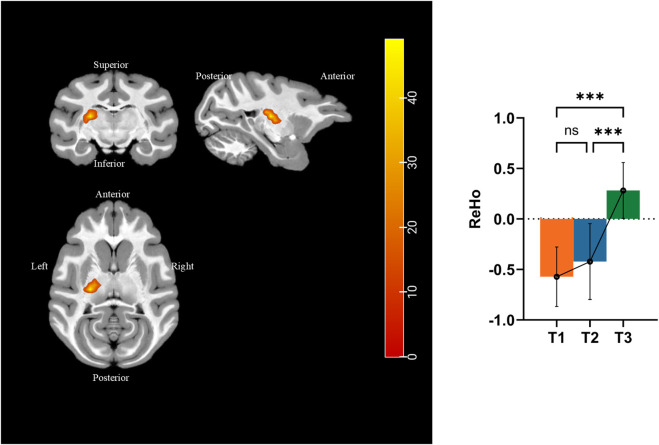
Different brain areas in ReHo among pre-HDBR and 21 and 42 days post-HDBR and *post hoc* analysis. ***, P < 0.001; ns, no significant; T1, pre-HDBR; T2, 21 days of HDBR; T3, 42 days of HDBR. **(A)** Group difference in FA (T2 < T1). **(B)** Group difference in MD (T2 < T1). **(C)** Group difference in AD (T2 < T1). **(D)** Group difference in RD (T2 < T1).

We performed ReHo analysis to identify the functional changes that occurred during simulated microgravity. Results were displayed in the T1 template from D99 rhesus atlas (Goldman-Rakic and Rakic, 1991). Figure 7 and Table 4 show that ReHo in the left thalamic reticular nucleus (RN) underwent significant changes. Compared to pre-HDBR, ReHo in the left thalamic reticular nucleus increased at 21 days of simulated microgravity and continued to increase by 42 days.

**TABLE 4:** Significant clusters displaying an alternation in ReHo after HDBR (voxel P < 0.001, cluster P < 0.05, P_FWE_ < 0.05).

Estimated area	Peak of mass			F Value	Cluster size (voxel)	Standardized ReHo		
	X	Y	Z			T1	T2	T3
L-RN	−11	−10.5	5	49.0041	16	−0.57 ± 0.29	−0.42 ± 0.38	0.28 ± 0.28

ReHo, regional homogeneity; L, left; RN, reticular nucleus.

## 4 Discussion

We performed longitudinal studies on the alterations in gray matter, white matter tracts, and resting-state functional activity during simulated microgravity in rhesus monkeys. Repeated sMRI, DTI, and fMRI scans were acquired at the baseline (3 days prior to HDBR) and 21 and 42 days after HDBR. We found GM abnormalities in the right substantia innominate (r-SI) of the BF area, right insula, left putamen (l-Pu), and left occipital gyrus. White matter fiber tracts were damaged in the left ILF, left FX, left corticospinal tract (CST), left SLF, left FAT, right UF, and bilateral IFO. Meanwhile, white matter in left SLF, left UF, middle cerebellar peduncle (MCP), bilateral FAT, bilateral ATR, and bilateral UF reinforced. White matter abnormality indicated that simulated microgravity influences the brain function in visuospatial awareness, motion, memory, emotion, execution, and language. ReHo increased in the left RN, indicating that local activity in the thalamic regions enhanced synchronously.

### 4.1 Gray matter changes

Structurally, gray matter is composed of neurons, axons, glial cells, and capillaries. Most studies attribute GM alterations to synaptogenesis, gliogenesis, neurogenesis, and vascular changes (Zatorre et al., 2012). We observed GM reduction in the left occipital gyrus, which changed after spaceflight, as observed in previous studies (Burles et al., 2023). Riascos et al. (2019) found a trend of decrease in the mean of the occipital cortical thicknesses after spaceflight. Koppelmans et al. (2016) showed that GM decreases were broadly distributed across the occipital lobes. Generally, occipital gyri process visual information and are an important part of the visual network. We found that simulated microgravity causes gray matter atrophy in the occipital gyrus, which may be related to decreased connectivity in certain spatial visual networks associated with this region. Similar to the actual spaceflight, the HDBR experiment led to a reduction in relatively monotonous visual stimuli, then reduced neural input, and redistribution of visual processing pathways (Ozdemir et al., 2018). This could be one of the reasons for gray matter atrophy in the occipital gyrus. When completing sensorimotor tasks under microgravity conditions, changes in visual and vestibular input alter astronauts' perception of moving objects, and the brain’s perception of motion relies on an egocentric spatial reference (Hao and Zhou, 2020). Consistent with previous results, the decrease in occipital gyrus GMV provides structural evidence that sensory reweighting affects sensorimotor performance during microgravity (Tays et al., 2021).

In addition, we observed an increase of GMV in the left putamen (Pu), consistent with a previous study reporting a similar trend (Riascos et al., 2019). Pu is part of the basal ganglia and is connected to the hippocampus, typically associated with motor control and learning. Neuroanatomical studies have shown that the posterior putamen is linked to the primary motor cortex and the supplementary motor area (SMA). The putamen plays a role in the initiation, execution, and regulation of voluntary movements. Moreover, the putamen is critical for stimulus–response associations, learning, or strategic planning, particularly in simpler, stereotyped behaviors such as habit formation and stimulus–response conditioning (Ja et al., 2008). In the HDBR experiment, rhesus monkeys showed reduced daily activity but could voluntarily perform eating and playing with toys. Our study found an increase in GMV in the left putamen, suggesting that even simple behavioral mechanisms require adaptation to simulated microgravity. On the one hand, the activity of anti-gravity muscle groups weakens, leading to diminished motor coordination and impaired balance and movement flexibility (Yang and Shen, 2003). On the other hand, the conditioned, habitual, and simple daily behaviors demand readaptation. So it is speculated that the putamen, a key node in the motor circuit, may play a crucial role in adapting motor capabilities and basic behavior patterns to microgravity (Hupfeld et al., 2022), potentially resulting in increased gray matter volume in the putamen.

This study also found an increase in GMV in the right substantia innominata (SI) region after 42 days of HDBR. The SI is part of the basal forebrain and contains the basal nucleus of Meynert, which may be involved in regulating various functions, including motor control, anger-related emotions, memory, and arousal. A mouse study identified the SI as a core region responsible for general aggressive responses (Zhu et al., 2021), linking it to anger-related behaviors. Meanwhile, in patients with Parkinson’s disease, cholinergic neuron loss and Lewy body formation were observed in the SI (Chen et al., 2024). Although previous studies on spaceflight or HDBR simulations did not report similar abnormalities in this brain region, the increased gray matter density here may be related to enhanced self-protective mechanisms in response to altered gravity conditions.

### 4.2 Fiber tract changes

We also characterized the development of white matter microstructure using DTI. Compared with the voxel-based analysis method in DTI, the TBSS method is better in image alignment and without the smoothing problem. The parameters FA, MD, AD, and RD reflect white matter integrity from different perspectives. FA is an indicator of water diffusion directionality and serves as a comprehensive measure of microstructural integrity. Reduced FA is typically associated with white matter injury, demyelination, or tract disarray (Alexander et al., 2007). MD represents the average rate of water diffusion in all directions within the brain tissue. Decreased MD suggests restricted water diffusion, which may result from increased cellular density or enhanced fiber tract integrity. AD and RD reflect water diffusion rates parallel and perpendicular to fiber tracts, respectively. AD reduction often correlates with axonal damage, where cellular necrosis leads to axonal degeneration and disintegration. The resulting cellular debris creates diffusion barriers parallel to axons, thereby lowering AD values in white matter (Alegiani et al., 2019). RD reduction indicates improved myelin integrity, increased axonal diameter/density, or perimyelinic edema, which restricts water diffusion perpendicular to fibers (Shekari and Nozari, 2023). This study observed significant decreases in FA, MD, AD, and RD during HDBR, suggesting that white matter alterations involve not only structural impairment but also concurrent myelin reinforcement and repair processes.

We found a decrease in the FA value in the left ILF and left FX after HDBR. These fiber tracts are primarily connected to the hippocampus, so their impairment may consequently lead to impaired hippocampal-related neurological functions, potentially resulting in declines in memory, emotion regulation, and executive functions. The ILF consists of long and short association fibers originating from the middle and inferior temporal gyri, parahippocampal gyrus, hippocampus, amygdala, and temporal pole. It connects the occipital cortex with the anterior temporal lobe and amygdala, terminating in the peristriate region (Maller et al., 2019). More than 40% of these fibers form hippocampal pathways, making them crucial for memory processes (Latini et al., 2017; Chen et al., 2020; Chen et al., 2022). As a part of the limbic system, the FX originates from the hippocampus and stretches to the diencephalon and basal forebrain (Kantarci, 2014). It plays a crucial role in emotion regulation and memory formation in cognitive processes. Our previous work proved that neurogenesis and proinflammatory mediators in the hippocampus could be affected in simulated microgravity (Lin et al., 2020). Although we found no alternation in the hippocampus by DTI, impairment of limbic fiber integrity may be associated with that of the hippocampus. After 42 days of HDBR, regions of reduced FA expanded but remained in the left ILF, suggesting that as the duration of microgravity exposure increases, the damage to hippocampal-related fiber connectivity progressively worsens.

Our findings also demonstrated AD reduction in the left SLF, left ILF, left CST, left FAT, right UF, and bilateral IFO in rhesus during HDBR, indicating that WM tract damage may cause dysfunction of spatial awareness, voluntary motion, emotion regulation, and language. Visuospatial orientation is a critical concern in microgravity research as impairments in these functions could pose significant risks to operational tasks in microgravity environments. The IFO, one of the major association tracts mediating visuospatial awareness (Voets et al., 2017; Herbet et al., 2017; Urbans et al., 2008), is a direct connection between ventral occipital and frontal regions. In some cases, a lesion in the IFO may contribute to neglect by impairing the top–down modulation of visual areas from the frontal cortex. As one of the longest association tracts in the brain, SLF connects the superior parietal to superior frontal lobes longitudinally along the dorsal premotor and dorsolateral prefrontal regions (Catani et al., 2002). SLF fibers adjacent to the superior parietal and frontal regions engage in the voluntary orientation of spatial attention through several networks (Wang et al., 2020), governing the working memory, integration, and attentional allocation of spatial information. The corticospinal tract (CST) is one of the most critical motor pathways in the central nervous system (Natali et al., 2025). Previous quantitative MRI studies on astronauts post-spaceflight revealed asymmetries in FA and MD within the bilateral corticospinal tracts (Hasan et al., 2018). In addition, an early study demonstrated increased excitability of the corticospinal tract during HDBR (Roberts et al., 2010). CST emerged as a primary focus for microgravity studies for controlling voluntary movements of the limbs and trunk. Impaired motor function in microgravity has been a persistent challenge in spaceflight, with countermeasures such as resistance training.

Additionally, white matter regions showed decreased MD and RD values after HDBR compared to pre-HDBR, including the bilateral ATR, bilateral FAT, bilateral UF, MCP, and left SLF tracts. These MD and RD reductions may be attributed to increased fiber density and enhanced connectivity within these tracts, which disturbed water molecule diffusion, particularly in the vertical direction to the axon. As a projection fiber linking the thalamus and prefrontal cortex, the ATR regulates cortical–subcortical interactions (Ayyıldız et al., 2023). Specifically, it mediates emotional regulation, reward-seeking behavior, and motivational processes, so its impairment represents a critical neural mechanism in psychiatric disorders like depression and bipolar disorder (Denier et al., 2020). The damage to the ILF and UF fiber tracts may contribute to emotional regulation dysfunction in microgravity environments. Simultaneously, we observed enhanced myelin integrity in the bilateral anterior thalamic radiation (ATR) fiber tracts, suggesting a potential shift in key neural hubs responsible for emotional modulation. FAT is implicated in specific executive functions, such as inhibitory control and conflict management and general motor regulation, as it is connected to the inferior frontal gyrus, pre-supplementary motor area, and subcortical structures (Dick et al., 2019). MCP, the primary fiber tract connecting the pons to the cerebellum, relays motor and cognitive information from the cerebral cortex to the cerebellum, with its core function being motor coordination (Jiang et al., 2020). The decreased MD and RD in MCP indicated enhanced myelination and increased axonal density. So the structural improvements may boost neural conduction efficiency, thereby augmenting its role in motor coordination. Although decreased MD and RD may reflect enhanced myelination or axonal density, an alternative explanation involves cytotoxic edema in glial cells. Such edema could compress axons and dendrites, physically obstructing water diffusion and lowering MD and RD values. Thus, further research is needed to clarify the molecular mechanisms underlying these observations.

### 4.3 Resting-state functional activity change

The fMRI results of this study revealed that simulated microgravity induces an increase in ReHo in the left RN of the thalamus. ReHo measures the temporal synchronization of BOLD signal fluctuations among neighboring voxels within a brain region, and the standardized ReHo reflects enhanced local functional synchronization, suggesting heightened neural activity or improved coordination in the region. Compared to pre-HDBR baseline levels, HDBR caused significant changes in ReHo values in the left RN region, with these changes continuing over prolonged HDBR exposure. RN is a thin layer of gray matter located between the lateral thalamic nuclei and the internal capsule. RN suppresses thalamic excitatory neurons via GABA release, thereby serving as a gatekeeper to regulate rhythmic activity in the thalamus and cortex. This inhibitory control enables the RN to modulate thalamus-related behavioral processes, particularly attention regulation, sleep–wake cycles (Barthó et al., 2014), and social memory (Feidi et al., 2024). Similar to a previous 72-h HDBR study (Liao et al., 2012), the thalamus has been recognized as a key target for neural alterations under microgravity. In our study, the enhanced local synchronization in the RN may reflect compensatory upregulation of inhibitory control within thalamus-related neural circuits under simulated microgravity. This could represent an adaptive mechanism to counteract disrupted sensory–motor integration or altered thalamocortical dynamics in a gravity-deprived environment.

### 4.4 Dynamic changes

Microgravity is a major stressor in the brain and produces significant effects in sensorimotor and vestibular brain regions (Willey et al., 2021). Previous evidence that prolonged microgravity leads to a progressive loss in cognitive function is sparse. The inability to obtain in-flight test from astronauts prevents observation of dynamic changes, thus creating a critical gap in longitudinal studies within this field. This study first reported dynamic brain changes in rhesus simulated microgravity models through MRI. The gray matter, white matter, and regional functional activity changes were mostly aggravated with time variation. The *post hoc* analysis of these changes suggested that significant changes are likely to occur at an earlier stage of simulated microgravity in rhesus monkeys. HDBR experiments on human volunteers showed that HDBR implied self-adaption or compensatory mechanisms to meet complex demands in cognitive flexibility, spatial navigation, and motor control functions (Zeng et al., 2016; Lia et al., 2015). Microgravity induces changes in the quantity and morphology of cortical synapses (DeFelipe et al., 2002) and neurogenesis in the hippocampus (Zhang et al., 2019), suggesting that Earth’s gravity is a necessary environment for normal synaptic development (Mao et al., 2024). Thus, our findings provide new insights into the effects of microgravity on the central nervous system and suggest potential neuroplastic mechanisms. In addition, we highlight significant microgravity-induced changes in visuospatial and motor-related brain regions in rhesus monkeys, involving gray matter volume, white matter integrity, and functional activity.

Moreover, we first synchronously reported the gray matter, white matter, and regional functional activity changes at the voxel-wise level. The study revealed that structural and functional changes under simulated microgravity occurred particularly in the brain regions associated with visuospatial cognition and motor control. Visuospatial cognition refers to the ability to perceive and analyze visual and vestibular information to determine the position, orientation, distance, and spatial relationships of objects or oneself. A study on astronauts found that spaceflight reduces neural connectivity between the occipital gyrus and other brain regions during spatial working memory (SWM) tasks (Salazar et al., 2023). Roberts et al. found that white matter regions emerged as significant predictors of altered visuospatial cognition (Roberts et al., 2019). We found that GMV of the left occipital gyrus significantly decreased, while the white matter integrity of the bilateral IFO was impaired. These changes may be linked to restricted visual information processing and vestibular dysfunction in microgravity, potentially leading to visuospatial deficits. Additionally, enhanced white matter fibers in the left SLF were observed at 21 days of simulated microgravity, suggesting possible compensatory mechanisms for visuospatial orientation. However, this enhancement was not detected at 42 days; instead, impaired white matter connectivity was observed in the SLF, indicating progressive fiber tract damage with prolonged microgravity exposure. Notably, previous DTI studies on astronauts frequently reported impairment of occipital-related white matter tracts (Riascos et al., 2019), whereas similar changes in SLF and IFO tracts were not observed. The HDBR experiment imposed more direct challenges to visuospatial orientation, so we may potentially enhance the sensitivity for detecting microgravity-induced effects on visuospatial abilities. These neuroplastic changes may serve as a critical strategy for adapting to abnormal visual–vestibular integration in microgravity.

Motor function, which involves the extrapyramidal system (for motor coordination), cerebellar circuits (for balance and precision), and the pyramidal tract (for voluntary movement), also exhibited adaptive changes. The study detected increased GMV in the putamen, enhanced white matter integrity in the MCP, and strengthened functional activity in the left reticular nucleus of the thalamus, suggesting adjustments in movement precision and coordination to adapt to the altered gravitational environment. However, white matter damage in the CST was observed, possibly compensating for unilateral impairment. Reduced limb activity and diminished gravitational loading have led current research to primarily attribute post-microgravity motor decline to muscle atrophy and bone loss. The findings further suggest that reasons of motor deficits in microgravity are due to factors beyond muscle atrophy. Such complex neural reconnection could hinder motor recovery, highlighting the need for integrated neuromuscular and neurostructural rehabilitation strategies.

### 4.5 Limitations

We found alternation in gray matter, white matter, and functional activity at the voxel-wise level after HDBR; however, there are still some limitations. The study’s small sample size might have biased our results, and non-human primates were used as the subjects. Our preliminary results need to be verified with more data in the future. Comparing research on large animal models with changes observed in astronauts or terrestrial analogs can help clarify whether animal studies could be an alternative scheme. The study’s self-controlled design limits our ability to determine whether the observed changes result specifically from simulated microgravity or co-occurring physiological alterations inherent to the HDBR model. In particular, the effects of immobilization itself may lead to decreased gray matter volume and white matter integrity changes in motor-related regions (Langer et al., 2012; Opie et al., 2016). Furthermore, repeated anesthesia may influence brain cerebral blood flow and neural connectivity (Li et al., 2021; Ma et al., 2019). Future studies incorporating a horizontal bed rest control group would be valuable to isolate more specific effects. Moreover, comprehensive experimental designs are required to determine the exact role of these structural changes. Behavior tests and cellular tests could be used to verify the relationship between the neurological function and MRI-based changes.

## 5 Conclusion

The brain’s adaptation to microgravity results from synergistic regulation of gray matter, white matter, and functional networks (Seidler et al., 2024). However, inconsistent findings currently preclude definitive evidence for such coordination. Our results suggested that monkey models of microgravity can be related to regionally specific alternations in gray matter, fiber tract, and neural activity. Some of these changes reflect damage to brain tissue and neural connections, while others involve reconstruction and reconnection. Generally, the subtle but significant MRI-based changes showed that rhesus monkeys’ brains require greater neuronal recruitment to maintain normal performance under simulated microgravity. The findings underlined the brain’s compensatory plasticity in adapting to the unique challenges of microgravity, offering critical implications for understanding neural adaptation in spaceflight. These findings may also provide valuable insights for human research. We hope that our results can improve the understanding of microgravity mechanisms and provide valuable references and guidance for future research and diagnosis.

## Data Availability

The raw data supporting the conclusions of this article will be made available by the authors, without undue reservation.
